# Sugar-Sweetened Beverage Consumption and Risks of Obesity and Hypertension in Chinese Children and Adolescents: A National Cross-Sectional Analysis

**DOI:** 10.3390/nu9121302

**Published:** 2017-11-30

**Authors:** Zhao-Huan Gui, Yan-Na Zhu, Li Cai, Feng-Hua Sun, Ying-Hua Ma, Jin Jing, Ya-Jun Chen

**Affiliations:** 1 Department of Maternal and Child Health, School of Public Health, Sun Yat-sen University, Guangzhou 510080, China; guizhh1991@163.com (Z.-H.G.); zhuyn3@mail.sysu.edu.cn (Y.-N.Z.); caili5@mail.sysu.edu.cn (L.C.); jingjin@mail.sysu.edu.cn (J.J.); 2 Division of Birth Cohort Study, Guangzhou Women and Children’s Medical Center, Guangzhou Medical University, Guangzhou 510623, China; 3 Department of Health and Physical Education, The Education University of Hong Kong, Tai Po 99907, Hong Kong, China; fhsun@eduhk.hk; 4 Institute of Child and Adolescent Health, School of Public Health, Peking University, Beijing 100191, China

**Keywords:** sugar-sweetened beverage, obesity, hypertension, child, adolescent

## Abstract

We investigated the consumption of sugar-sweetened beverage (SSB) and its association with obesity and hypertension in a national sample of children and adolescents in China, where many low- and middle-income families live. Data were obtained from a 2014 national intervention program against obesity in Chinese children and adolescents aged 6–17 years. Height, weight, waist circumference, and blood pressure were measured. Information of SSB consumption, socioeconomic status, dietary intake, screen time, and physical activity were self-reported. Multivariate logistic regression was used to assess the association of SSB consumption with obesity and hypertension. A total of 66.6% of the 53,151 participants reported consuming SSB. The per capita and per consumer SSB intake were 2.84 ± 5.26 servings/week and 4.26 ± 5.96 servings/week, respectively. Boys, older children, and adolescents, and individuals with long screen time or high physical activity or low parental education level were more likely to consume SSB. Participants who were high SSB consumers had a higher odds ratio (1.133, 95% CI: 1.054–1.217) than non-consumers for having abdominal obesity after adjustment for age, sex, residence, socioeconomic status, diet, screen time, and physical activity. However, SSB consumption was not associated with general obesity or hypertension in children and adolescents. In conclusion, more than half of the children and adolescents in China consumed SSB, which was independently related to a high risk of abdominal obesity. The results of this study indicated that SSB reduction strategies and policies may be useful in preventing obesity among Chinese children and adolescents.

## 1. Introduction

The global incidence of childhood obesity has nearly doubled in the past 30 years [1]. In China, a quarter of children aged 7–18 years were classified as overweight or obese in 2010 [2]. Chinese children have also experienced a significant increase in blood pressure in recent years [3]. Childhood obesity and hypertension may persist through adulthood [4,5], and both are associated with an increased risk of cardiovascular disease or diabetes [6,7]. Epidemiological evidence suggests that dietary intake, physical activity, and socioeconomic factors are highly correlated with the initiation and development of childhood obesity and elevated blood pressure [8]. Among dietary patterns, sugar-sweetened beverage (SSB) consumption has elicited considerable interest because of its relation to high calorie intake and metabolic disorders [9,10].

The US Nutrition Examination Survey showed that nearly 64% of the youth aged 2–19 years consume SSB daily [11]. These children consume an average of 155 kcal, which contribute 8.4% of the daily energy intake [11]. In Australia, 46.7% of the children aged 2–18 years drink SSB, and the youth consume an average of 217 mL of SSB per day, which is equivalent to 5.5% of their total energy intake [12]. In Mexico, SSB are the main sources of added sugar intake [13], contributing 8.3% of the total energy intake in school-aged children and adolescents [14]. These figures have exceeded the recommended intake, which was proposed by the World Health Organization (<5% energy from free sugars for additional health benefits) [15]. However, only a few studies have investigated SSB consumption among children in China, where many low- or middle-income families reside [16,17]. One such study conducted in 2010 merely described the types and prevalence of SSB intake among urban school children aged 6–13 years [16]. Meanwhile, our previous study focused only on children living in Southern China [17]. Thus, a nationally-representative study that considers the amount, frequency, and prevalence of SSB consumption by Chinese children and adolescents is required. 

A growing body of observational evidence suggests that SSB consumption may parallel the increase in blood pressure and the risk of obesity among children [18,19,20]. Proposed reasons for these associations include increased sodium intake [19], alteration of serum uric acid levels [21], or dysregulation of satiety and caloric compensation [22]. Conversely, no such association was observed in some other studies in children and adolescents [16,23,24,25]. Further investigation of this relationship in terms of the consistency of related studies is necessary. 

Many factors that influence SSB consumption, such as socioeconomic status, behavioral factors, and dietary factors, are typically contextualized within a socioecological framework at the individual, social, and environmental (both micro- and macro-) levels. For example, several studies indicated that children with low family income [26], reduced milk intake [27], or long screen time [28] are vulnerable to diets with high SSB. Therefore, a comprehensive examination is needed to gain insight into the effects of these influencing factors on the SSB consumption of Chinese children. Such information may provide opportunities for reducing SSB intake and, thus, prevent adulthood obesity, hypertension, and other associated cardiovascular diseases in the future.

This study aims to describe the status of SSB consumption and examine the association between SSB consumption and the risks of obesity and hypertension in a nationally representative sample of Chinese children and adolescents aged 6–17 years.

## 2. Methods

### 2.1. Subjects

The data were collected from a 2013 national multicenter intervention program against obesity in Chinese children and adolescents [29]. The general methodology has already been published [29]. This study was approved by the Ethical Committee of Peking University, and all participants provided their informed consent. The study involves a nationally representative sample of children and adolescents aged 6–17 years who were selected based on a random sampling of more than 60,000 subjects. The final sample consisting of 53,151 participants completed questionnaires and anthropometric measurements, and the response rate was 88%.

### 2.2. Data Collection

Demographic information (age, sex, and residence), SSB consumption, diet condition, screen time, and physical activity were obtained through a self-reported questionnaire for children. Parental education and family income were collected through a self-reported questionnaire for parents. Each participant was required to answer two questions on the frequency of consumption and usual portion size (one serving = 250 mL) of all SSB, such as Coca-Cola, Sprite, orange juice, Nutrition Express, and Red Bull. These items were combined to estimate the weekly SSB consumption (servings/week), and two variables (SSB servings and SSB frequency) were used to describe the SSB consumption status. The distribution of SSB intake was highly skewed, and transformation of data was not feasible because of the large number of participants who reported zero intake. Therefore, SSB serving and SSB frequency were categorized by using tertile cutoffs as follows: 0 servings/week, 0–2 servings/week, >2 servings/week; 0 times/week, 1–2 times/week, and >2 times/week.

Diet milk, meat, and fried food were separately assessed with three questions (how often did you consume milk/meat/fried food in the past week?), and the responses were categorized into two levels (low and high, times/week) by using dichotomy cutoffs. The screen time factor referred to the total time spent watching TV, playing computer or video games, and using the Internet. The screen time variable was also dichotomized (<2 h/day or ≥2 h/day) in accordance with the guidelines of the American Academy of Pediatrics [30]. In addition, children reported frequency and duration of vigorous-intensity physical activities (e.g., running, basketball, football, and swimming), moderate-intensity physical activities (e.g., cycling, table tennis, badminton, and calisthenics), and walking in the past seven days. The responses were converted into metabolic equivalent task (MET)-minutes per week by multiplying the MET level of each activity by its frequency and duration (MET minutes/week) [31]. The calculated values of all such activities were then summed to determine the overall physical activity. The participants were classified into three categories according to the tertiles of their overall physical activity. Socioeconomic status was calculated according to the three items in the parental questionnaire (paternal education, maternal education, and family income). Paternal or maternal education was divided into four groups, namely, primary school or below, junior high school, high school, and junior college or above. Family income was divided into five groups on the basis of the “household monthly income” item of this study and subsequently defined as 2000 RMB/month or below, 2000–5000 RMB/month, 5000–8000 RMB/month, 8000 RMB/month or above, and do not know or no answer.

Body mass, height, waist circumference, and blood pressure were measured in accordance with standard protocols [29]. BMI (body mass index) was calculated as body mass (kg) divided by the square of body height (m^2^). Overweight and obesity were defined on the basis of the age- and gender-specific BMI cutoffs recommended by the Working Group of Obesity in China [32]. Waist circumference ≥90th percentile for their age and sex was considered a case of abdominal obesity in accordance with the Chinese children percentile [33]. Systolic blood pressure (SBP)/diastolic blood pressure (DBP) levels were categorized into three groups, namely, <90th percentile (normal pressure); ≥90th and <95th percentile (pre-hypertension); and ≥95th percentile (hypertension) for their age and sex [34].

### 2.3. Statistical Analyses

Statistical analyses were completed with SPSS 21.0 (IBM, Armonk, NY, USA). All statistical significance tests were two-sided, and a level of α < 0.05 was considered statistically significant. The chi-square test was used to analyze qualitative variables, and a comparison of the mean of quantitative variables was conducted through an analysis of variance (ANOVA). To adjust the relationships for possible confounders, multinomial or binary logistic regression models were used to examine the odds ratio (OR) of the general and abdominal obesity or hypertension across the SSB categories in four models, namely, Model 1: crude model (without adjustment); Model 2: adjusted for age, sex, and residence; Model 3: additionally adjusted for maternal education, paternal education, family income, screen time, and physical activity; and Model 4: additionally adjusted for meat and fried food for obesity and meat, fried food, height, and BMI for hypertension.

### 2.4. Ethics

The study was conducted in accordance with the Declaration of Helsinki and approved by the Ethical Committee of the Peking University. Written informed consent was obtained from both children and adolescents and their legal guardians prior to their participation in the study.

## 3. Results

**Figure 1:**
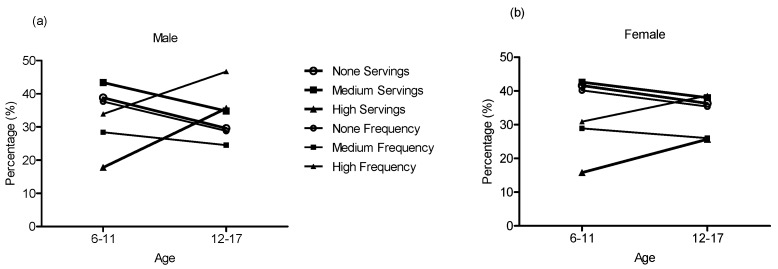
Sugar-sweetened beverage consumption of boys (**a**) and girls (**b**).

Table 1 and Figure 1 show the status of SSB consumption according to age and sex. Overall, 66.6% of all participants consumed SSB (69.7% for boys and 63.3% for girls), and 9.6% (12.4% for boys and 6.7% for girls) reported a consumption of ≥7 servings of SSBs per week. The per capita and per consumer SSB consumption were 2.84 ± 5.26 servings/week (~0.41 servings/day) and 4.26 ± 5.96 servings/week (~0.61 servings/day), respectively. Boys presented a higher SSB consumption than girls (*p* < 0.05). The trend showed that both boys and girls exhibited increasing SSB consumption with age (*p* < 0.001).

The characteristics of participants according to their SSB consumption are described in Table 2. Children who were non-consumers of SSB had parents with higher levels of education and lower physical activity and were less likely to have ≥2 h of screen time daily than those in the medium and high SSB consumption categories (all *p* < 0.05). Moreover, non-consumers presented lower BMI, waist circumference, SBP, and DBP (all *p* < 0.05) than consumers. For dietary intake, non-consumers had increased intake of milk, but decreased intake of meat, high-energy food, and fried food (all *p* < 0.05).

Table 3 displays the distribution of the prevalence of general and abdominal obesity and hypertension by SSB consumption. As shown, SSB consumption was associated with general and abdominal obesity, SBP, and hypertension (all *p* < 0.05). However, SSB consumption showed no relationship with DBP.

Table 4 summarizes the associations of socioeconomic status, dietary intake, and screen time with the prevalence of general and abdominal obesity and hypertension. Neither general obesity nor abdominal obesity was associated with diets, including meat and fried food. Parental education, family income, and screen time were related to general and abdominal obesity and hypertension (all *p* < 0.05).

Table 2 and Table 4 show that parental education, family income, meat, fried food, screen time, and physical activity were associated with SSB consumption and/or anthropometric variables. Therefore, these factors may be adjusted when evaluating the relationships between SSB consumption and the risks of anthropometric variables.

The OR for general and abdominal obesity and hypertension across the SSB consumption categories in the four models are listed in Table 5. First, without considering confounding factors, the risks of overweight, abdominal obesity, and pre-hypertension in the high servings of SSB were higher than those of non-consumers; the OR were 1.085 (95% CI: 1.016–1.157), 1.107 (95% CI: 1.049–1.168), and 1.472 (95% CI: 1.380–1.570), respectively. An unadjusted logistic regression analysis indicated that children consuming medium servings of SSB have a higher risk of being overweight compared with the non-consumers (OR: 1.070, 95% CI: 1.006–1.138). Second, the association of SSB consumption and abdominal obesity remained significant after adjustment for age, sex, residence, parental education, family income, screen time, physical activity, meat, and fried food. A 13.3% increased risk was observed for high servings of SSB (OR: 1.133, 95% CI: 1.054–1.123) compared with that for non-consumers.

## 4. Discussion

To the best of our knowledge, this study is the first to explore the status of SSB consumption and its association with obesity and hypertension in a large nationally representative sample of Chinese children and adolescents aged 6–17 years. Overall, 66.6% of the children consumed SSB. The per capita and per consumer SSB intake was 2.84 servings/week (~0.41 servings/day) and 4.26 servings/week (~0.61 servings/day). Boys and older adolescents consumed a large amount of SSB. SSB consumption was positively associated with abdominal obesity after adjustment for age, sex, residence, parental education, family income, screen time, physical activity, meat, and fried food. Neither general obesity nor hypertension showed an association with SSB consumption. 

Our findings provide a recent picture of the SSB consumption of a nationally representative sample of Chinese children and adolescents. A relatively high percentage (66.6%) of Chinese children and adolescents consumed SSB, although the intake amount per capita/per consumer was relatively low. This finding is consistent with a previous study conducted on younger children aged 3–7 years in China [35], but it contradicts the results of studies on children from developed countries [12]. For example, in Australia the prevalence of SSB intake was not high, although the daily amount was twice that of Chinese children reported in the present study [12]. The prevalence of SSB consumption in the present study revealed a marked increase from the SSB consumption level reported by a 2010 survey in China, which found that only 46% of children consumed SSB [16]. This phenomenon is in contrast to the situations in the United States and in Australia, where SSB consumption by children and adolescents have been reduced in the past several decades [11,12]. The driving force behind this upward trend in SSB intake likely included the growth of household incomes in China, the attempts of the food industry to attract customers, or the shift in the patterns of caloric beverage intake. Nonetheless, Chinese children have a substantially lower consumption level than children in developed countries [11,12]. In the present study, the per capita and per consumer SSB intake was only 0.41 servings/day (103 mL/day) and 0.61 servings/day (153 mL/day), and only 9.6% of children had ≥1 SSBs consumption per day. By contrast, in the United States, more than 64% of the youth aged 2–19 years had at least 1 SSB intake daily [11]. In Australia, the total volume of consumed SSBs reached 217 mL/day across all children and 355 mL/day per consumer [12]. The potential reason for this regional variation is the difference among food environments, such as access to food and beverages, advertising, culture, and food regulations.

Consistent with other findings [36], adolescents and older children consumed more SSBs than the younger children in the present study. A longitudinal study showed that [37] milk and fruit juice intake decreased from early childhood to late adolescence with an increase in SSB consumption. These data indicated that the high SSB consumption at late age ranges influence the subsequent dietary intake of children. Thus, timely intervention that discourages early consumption of SSB is important. Furthermore, girls are less likely to consume SSB than boys [38] presumably because girls in China are more focused on their physical appearance and diet.

After adjustment for the important potential confounders, SSB consumption remained associated with a high risk of abdominal obesity. This finding is similar to the result of a previous study on Tehranian children [39]. In addition, such a positive association was also observed in Chinese children in previous research [16,17]. Several possible mechanisms may explain the adverse effects of SSB intake on abdominal obesity. Sugar, when added in liquid form, is less satiating than when added in solid form and, thus, contributes to incomplete energy compensation and ultimately adds to the total calories consumed [22]. Furthermore, SSB consumption may be a marker of a generally poor-quality diet and behavior. Notably, children consuming large servings of SSB were more likely to have ≥2 h of screen time, reduced milk intake, and high consumption of meat and high-energy diet, as shown in previous studies [19,28]. Moreover, residual confounders persisted in the observational studies despite the statistical adjustments for these confounders. Thus, the relationship reported in the present study might be a result of the overall clustering of “unhealthy” diet and sedentary behaviors.

Although SSB consumption was not positively associated with general obesity in the present study, a growing body of evidence supports this notion [18,19,22]. A prospective study conducted in the United States found that the OR for becoming obese in childhood increases 0.6 times for each additional serving of SSB [22]. The combined mean estimate of SSB intake in that study [22] was considerably greater than those of children participating in the present study. The lack of a significant association between SSB and general obesity might be related to the low levels of SSB consumption of Chinese children and adolescents in the present study. Convincing studies in adults suggest that SSB consumption was associated with hypertension [40,41], however, the evidence for children and adolescents were inconsistent [16,25]. Similar to two other previous studies [16,25], our analysis did not find such a relationship. The limited amount of SSB consumption of the Chinese youth might be the reason for the non-significant result.

The present study presents several limitations. First, causation could not be inferred because the study applied a cross-sectional analysis. The specific influence of SSB consumption on the risk of abdominal obesity could not be determined with certainty. Second, the diet questionnaire failed to avoid recall bias and is, thus, likely to be an underestimation of the true value of SSB consumption. Nonetheless, this study has several strengths, including the large nationally-representative sampling design stratified by geographical location, age, and socioeconomic status; the comprehensive collection of dietary intake, anthropometric, and demographic data; and the good response rate of the participants. The findings of this study provide a timely update on the current status of SSB consumption among Chinese children. From a public health standpoint, SSB consumption should be reduced to prevent abdominal obesity by performing various actions, such as changing the advertising of SSBs or controlling the access to SSBs in schools or other environments.

## 5. Conclusions

Although SSB consumption was common among the evaluated Chinese children and adolescents, they reported low levels of SSB consumption. Older children and boys were more likely to drink SSBs. Furthermore, SSB consumption was associated with increased risk of abdominal obesity regardless of parental education, family income, screen time, physical activity, meat and fried food intake. However, SSB consumption was not related to general obesity and hypertension. Public health practitioners and parents should encourage children to consume calorie-free beverages, such as water instead of SSB to avoid an excessive waist circumference. This step may mitigate the small, but important, contribution of SSBs to the current epidemic of childhood abdominal obesity.

## Figures and Tables

**Table 1:** SSB intake of the participants.

Age (Years)	Average (Servings/Day) (Mean ± SD)			Servings/Day (%)			Average (Frequency/Week) (Mean ± SD)			Frequency/Week (%)		
	Total	Boys	Girls	None Servings	Medium Servings	High Servings	Total	Boys	Girls	None Frequency	Medium Frequency	High Frequency
6–11	1.51 ± 2.81	1.59 ± 2.86	1.43 ± 2.76 *	40.1	43.0	16.8	1.23 ± 1.48	1.27 ± 1.50	1.19 ± 1.45	38.9	28.6	32.5
12–17	2.95 ± 5.41	3.56 ± 6.24	2.32 ± 4.30 *	32.8	36.4	30.8	1.28 ± 1.51	1.36 ± 1.55	1.21 ± 1.46 *	37.5	28.8	33.7
Total	2.84 ± 5.26	3.40 ± 6.06	2.25 ± 4.20 *	33.4	36.9	29.7	1.62 ± 1.78	1.80 ± 1.89	1.43 ± 1.63 *	32.6	25.5	41.9

* Boys vs. girls, *p* < 0.05.

**Table 2:** Basic characteristics of the participants according to SSB intake.

Variables	None Servings/Week	Medium Servings/Week	High Servings/Week	*p*-Value	None Frequency/Week	Middle Frequency/Week	High Frequency/Week	*p*-Value
Residence, *n* (%)				0.981				0.04
Urban	11,209 (33.4)	12,384 (36.9)	9957 (29.7)		11,209 (32.5)	8947 (26.0)	14,311 (41.5)	
Rural	6564 (33.5)	7231 (36.9)	5806 (29.6)		6564 (32.7)	4964 (24.7)	8545 (42.6)	
Paternal education, *n* (%)				<0.001				<0.001
Primary school or below	1180 (34.6)	1183 (34.7)	1050 (30.7)		1180 (33.7)	889 (25.4)	1433 (40.9)	
Junior high school	5481 (31.7)	6441 (37.2)	5389 (31.1)		5481 (30.8)	4531 (25.5)	7783 (43.7)	
High school	4256 (32.3)	5024 (38.1)	3908 (29.6)		4256 (31.4)	3515 (26.0)	5770 (42.6)	
Junior college or above	5278 (38.2)	5204 (37.7)	3317 (24.1)		5278 (37.5)	3706 (26.3)	5102 (36.2)	
Maternal education, *n* (%)				<0.001				<0.001
Primary school or below	1579 (33.6)	1680 (35.7)	1443 (30.7)		1579 (32.6)	1237 (25.5)	2033 (41.9)	
Junior high school	5709 (32.0)	6586 (36.9)	5553 (31.1)		5709 (31.2)	4556 (24.9)	8056 (43.9)	
High school	4059 (32.5)	4803 (38.5)	3620 (29.0)		4059 (31.7)	3404 (26.6)	5333 (41.7)	
Junior college or above	4792 (38.1)	4764 (37.9)	3022 (24.0)		4792 (37.3)	3418 (26.6)	4647 (36.1)	
Family income, RMB/month, *n* (%)				0.037				0.001
2000 or below	1194 (36.1)	1179 (35.7)	932 (28.2)		1194 (35.3)	874 (25.8)	1318 (38.9)	
2000–5000	3635 (33.4)	4105 (37.7)	3138 (28.9)		3635 (32.5)	3033 (27.2)	4500 (40.3)	
5000–8000	2744 (33.0)	3188 (38.3)	2395 (28.7)		2744 (32.2)	2255 (26.4)	3529 (41.4)	
8000 or above	3175 (34.4)	3545 (38.5)	2499 (27.1)		3175 (33.7)	2408 (25.6)	3829 (40.7)	
Don’t know or no answer	4624 (35.3)	4859 (37.1)	3626 (27.6)		4624 (34.3)	3341 (24.8)	5501 (40.9)	
Screen time, *n* (%)				<0.001				<0.001
<2 h	11,631 (37.0)	11,874 (37.8)	7940 (25.3)		11,631 (36.1)	8381 (26.0)	12,195 (37.9)	
≥2 h	3669 (24.5)	5409 (36.2)	5884 (39.3)		3669 (24.5)	3739 (24.4)	7909 (51.6)	
Physical activity, MET (min/week), Mean ± SE	3064.3 ± 28.9	3289.1 ± 27.3	3805.9 ± 37.5	<0.001	3064.3 ± 28.9	3343.6 ± 34.0	3624.6 ± 29.0	<0.001
Dietary consumption (times/week), Mean ± SE								
Fruit	1.28 ± 0.01	1.23 ± 0.01	1.33 ± 0.01	<0.001	1.28 ± 0.01	1.26 ± 0.01	1.29 ± 0.01	0.037
Vegetables	1.87 ± 0.01	1.72 ± 0.01	1.81 ± 0.01	<0.001	1.87 ± 0.01	1.77 ± 0.01	1.76 ± 0.01	<0.001
Meat	4.91 ± 0.02	4.89 ± 0.02	5.24 ± 0.02	<0.001	4.91 ± 0.02	4.79 ± 0.02	5.19 ± 0.02	<0.001
Milk	4.61 ± 0.02	4.57 ± 0.02	4.37 ± 0.02	<0.001	4.61 ± 0.02	4.49 ± 0.02	4.46 ± 0.02	<0.001
High-energy food	1.32 ± 0.01	1.99 ± 0.01	2.80 ± 0.01	<0.001	1.32 ± 0.01	1.80 ± 0.01	2.66 ± 0.01	<0.001
Fried food	0.68 ± 0.01	1.18 ± 0.01	1.88 ± 0.01	<0.001	0.68 ± 0.01	1.06 ± 0.01	1.73 ± 0.01	<0.001
Anthropometry, Mean ± SE								
BMI (kg/m^2^)	18.24 ± 0.03	18.39 ± 0.03	19.36 ± 0.03	<0.001	18.24 ± 0.03	18.32 ± 0.03	19.08 ± 0.03	<0.001
Waist circumference (cm)	63.65 ± 0.08	64.15 ± 0.08	67.59 ± 0.09	<0.001	63.65 ± 0.08	63.92 ± 0.09	66.62 ± 0.07	<0.001
Hip circumference (cm)	75.61 ± 0.09	76.01 ± 0.08	80.85 ± 0.10	<0.001	75.61 ± 0.09	75.72 ± 0.10	79.47 ± 0.08	<0.001
SBP (mmHg)	103.44 ± 0.09	103.91 ± 0.09	106.23 ± 0.10	<0.001	103.44 ± 0.09	103.71 ± 0.10	105.56 ± 0.08	<0.001
DBP (mmHg)	65.80 ± 0.07	65.90 ± 0.06	67.04 ± 0.07	<0.001	65.80 ± 0.07	65.79 ± 0.07	66.73 ± 0.06	<0.001

SSB, sugar-sweetened beverage; MET, metabolic equivalent task; BMI, body mass index; SBP, systolic blood pressure; DBP, diastolic blood pressure.

**Table 3:** Distribution of obesity and blood pressure (%) by SSB intake.

Characteristics	Total *n* (%)	None Servings/Week	Medium Servings/Week	High Servings/Week	None Frequency/Week	Middle Frequency/Week	High Frequency/Week
BMI							
Underweight, *n* (%)	4284 (6.9)	1262 (7.1)	1282 (6.6)	1040 (6.6)	1262 (7.1)	925 (6.6)	1485 (6.5)
Normal, *n* (%)	43,607 (69.8)	12,392 (69.7)	13,581 (69.2)	10,905 (69.2)	12,392 (69.7)	9578 (68.9)	15,906 (69.6)
Overweight, *n* (%)	8000 (12.8)	2214 (12.5)	2596 (13.2)	2113 (13.4)	2214 (12.5)	1845 (13.3)	3032 (13.3)
Obesity, *n* (%)	6626 (10.6)	1905 (10.7)	2156 (11.0)	1705 (10.8)	1905 (10.7)	1563 (11.2)	2433 (10.6)
*p*-value			0.009			0.009	
Abdominal obesity							
No, *n* (%)	44,425 (78.1)	12,520 (78.4)	13,715 (77.6)	11,461(76.7)	12,520 (78.4)	9224 (77.6)	15,952 (76.9)
Yes, *n* (%)	12,460 (21.9)	3443 (21.6)	3962 (22.4)	3489 (23.3)	3443 (21.6)	2659 (22.4)	4792 (23.1)
*p*-value			0.001			0.002	
DBP							
Normal, *n* (%)	52,623 (84.5)	15,046 (85.1)	16,532 (85.2)	13,241 (84.3)	15,046 (85.1)	11,805 (85.2)	19,267 (84.6)
Pre-hypertension, *n* (%)	4902 (7.9)	1275 (7.2)	1469 (7.5)	1494 (9.5)	1275 (7.2)	991 (7.2)	2055 (9.0)
Hypertension, *n* (%)	4721 (7.6)	1367 (7.7)	1431 (7.3)	968 (6.2)	1367 (7.7)	1062 (7.7)	1442 (6.3)
*p*-value			0.282			0.713	
SBP							
Normal, *n* (%)	49,941 (80.3)	14,366 (81.2)	15,787 (80.8)	12,183 (77.6)	14,366 (81.2)	11,254 (81.2)	17,881 (78.6)
Pre-hypertension, *n* (%)	6697 (10.8)	1701 (9.6)	1953 (10.0)	2155 (13.7)	1701 (9.6)	1327 (9.6)	2901 (12.7)
Hypertension, *n* (%)	5585 (9.0)	1622 (9.2)	1790 (9.2)	1366 (8.7)	1622 (9.2)	1281 (9.2)	1977 (8.7)
*p*-value			<0.001			<0.001	
Hypertension							
Normal, *n* (%)	46,690 (75.1)	13,470 (76.2)	14,836 (76.0)	11,440 (72.9)	13,470 (76.2)	10,568 (76.3)	16,800 (73.9)
Pre-hypertension, *n* (%)	7633 (12.3)	1957 (11.1)	2213 (11.3)	2446 (15.6)	1957 (11.1)	1510 (10.9)	3288 (14.5)
Hypertension, *n* (%)	7856 (12.6)	2249 (12.7)	2464 (12.6)	1805 (11.5)	2249 (12.7)	1771 (12.8)	2654 (11.7)
*p*-value			<0.001			<0.001	

SSB, sugar-sweetened beverage; BMI, body mass index; SBP, systolic blood pressure; DBP, diastolic blood pressure.

**Table 4:** Association of socioeconomic status, diet, screen time, and physical activity with obesity and blood pressure.

	BMI, *n* (%)				Abdominal Obesity, *n* (%)		Hypertension, *n* (%)		
Variables	Underweight	Normal	Overweight	Obesity	No	Yes	Normal	Pre-Hypertension	Hypertension
Paternal education									
Primary school or below	260 (7.1)	2689 (73.0)	436 (11.8)	299 (8.1)	2874 (81.9)	637 (18.1)	2668 (72.8)	549 (15.0)	450 (12.3)
Junior high school	1193 (6.4)	13,163 (71.0)	2252 (12.2)	1922 (10.4)	13,593 (79.2)	3578 (20.8)	13,321 (72.2)	2599 (14.1)	2526 (13.7)
High school	905 (6.4)	9656 (68.3)	1908 (13.5)	1611 (11.8)	9750 (76.1)	3062 (23.9)	10,613 (75.6)	1611 (11.5)	1821 (13.0)
Junior college or above	1039 (7.1)	9653 (65.5)	2182 (14.8)	1862 (12.6)	9674 (74.7)	3268 (25.3)	11,652 (79.5)	1404 (9.6)	1601 (10.9)
*p*-value	<0.001				<0.001		<0.001		
Maternal education									
Primary school or below	364 (7.2)	3739 (73.7)	552 (10.9)	421 (8.3)	3936 (82.1)	860 (7.9)	3681 (72.9)	729 (14.4)	639 (12.7)
Junior high school	1242 (6.5)	13,472 (70.6)	2353 (12.3)	2012 (10.5)	13,958 (78.9)	3724 (21.1)	13729 (72.3)	2690 (14.2)	2576 (13.6)
High school	841 (6.3)	9010 (67.5)	1861 (13.9)	1636 (12.3)	9097 (75.4)	2969 (24.6)	10,059 (75.8)	1455 (11.0)	1761 (13.3)
Junior college or above	944 (7.0)	8848 (65.7)	1998 (14.8)	1686 (12.5)	8778 (74.5)	2998 (25.5)	10,704 (79.9)	1280 (9.6)	1411 (10.5)
*p*-value	<0.001				<0.001		<0.001		
Family income, RMB /month									
2000 or below	254 (6.5)	2843 (72.4)	448 (11.4)	380 (9.7)	2967 (79.8)	752 (20.2)	2759 (71.5)	542 (13.9)	572 (14.6)
2000–5000	833 (6.5)	8938 (69.8)	1642 (12.8)	1385 (10.8)	9349 (78.5)	2568 (21.5)	9238 (71.7)	1751 (13.7)	1849 (14.5)
5000–8000	621 (6.5)	6584 (68.7)	1261 (13.2)	1119 (11.7)	6708 (77.2)	1978 (22.8)	7044 (73.9)	1171 (12.3)	1318 (13.8)
8000 or above	685 (6.7)	6886 (67.4)	1457 (14.3)	1190 (11.6)	6834 (75.9)	2165 (24.1)	7975 (78.5)	1007 (9.9)	1173 (11.6)
Don’t know or no answer	1072 (7.2)	10,426 (69.6)	1874 (12.5)	1605 (10.7)	10,524 (77.9)	2987 (22.1)	11,245 (75.5)	1822 (12.2)	1835 (12.3)
*p*-value	<0.001				<0.001		<0.001		
Meat									
Low	1676 (6.3)	18,562 (69.9)	3346 (12.6)	2959 (11.1)	19,072 (77.6)	5519 (22.4)	19,129 (72.4)	3608 (13.7)	2806 (10.6)
High	2013 (7.1)	19,459 (68.9)	3775 (13.4)	2982 (10.6)	19,758 (77.7)	5676 (22.3)	21,858 (77.8)	3172 (11.3)	2359 (8.4)
*p*-value	0.128				0.734		<0.001		
Fried food									
Low	1617 (6.9)	16,174 (69.3)	3051 (13.1)	2485 (10.7)	16,465 (77.8)	4706 (22.2)	17,798 (76.7)	2649 (11.4)	2753 (11.9)
High	2022 (6.5)	21,515 (69.5)	4032 (13.0)	3409 (11.0)	22,015 (77.4)	6412 (22.6)	22,827 (74.0)	4098 (13.3)	3906 (12.7)
*p*-value	0.122				0.387		<0.001		
Screen time									
<2 h	2222 (6.9)	22,177 (68.6)	4353 (13.5)	3584 (11.1)	22,405 (76.7)	6820 (23.3)	24,428 (75.9)	3895 (12.1)	3857 (12.0)
≥2 h	973 (6.3)	10,655 (69.2)	1982 (12.9)	1792 (11.6)	11,206 (77.6)	3239 (22.4)	11,483 (74.9)	1933 (12.6)	1909 (12.5)
*p*-value	0.262				0.033		0.022		
Physical activity, MET (min/week)									
1413.0 or below	1085 (6.7)	11,186 (69.5)	2072 (12.9)	1750 (10.9)	11,082 (77.7)	3176 (22.3)	12,788 (79.9)	1721 (10.7)	1505 (9.4)
1413.0–3399.5	1117 (6.8)	11,320 (69.3)	2134 (13.1)	1765 (10.8)	11,577 (77.3)	3409 (22.7)	13,018 (80.0)	1782 (10.9)	1475 (9.1)
3399.5 or above	1047 (6.5)	11,156 (68.9)	2193 (13.5)	1795 (11.1)	11,888 (77.2)	3502 (22.8)	12,750 (79.7)	1879 (11.7)	1486 (9.2)
*p*-value	0.444				0.533		0.074		

BMI, body mass index; MET, metabolic equivalent task.

**Table 5:** Odds ratios (CI 95%) for general and abdominal obesity and hypertension across SSB intake.

	Overweight OR (95% CI)	Obesity OR (95% CI)	Abdominal Obesity OR (95% CI)	Pre-Hypertension OR (95% CI)	Hypertension OR (95% CI)
**SSB**					
**SSB Servings/week**					
*Model 1*					
None Servings	1	1	1	1	1
Medium Servings	1.070 (1.006–1.138) *	1.033 (0.966–1.103)	1.050 (0.998–1.106)	1.027 (0.962–1.096)	0.995 (0.935–1.058)
High Servings	1.085 (1.016–1.157) *	1.017 (0.948–1.091)	1.107 (1.049–1.168) ***	1.472 (1.380–1.570) ***	0.945 (0.884–1.010)
*Model 2*					
None Servings	1	1	1	1	1
Medium Servings	1.054 (0.990–1.121)	1.017 (0.951–1.088)	1.053 (1.000–1.109)	1.000 (0.935–1.069)	0.992 (0.933–1.056)
High Servings	1.052 (0.984–1.125)	1.057 (0.983–1.137)	1.124 (1.063–1.187) ***	1.052 (0.983–1.126)	0.975 (0.910–1.044)
*Model 3*					
None Servings	1	1	1	1	1
Medium Servings	1.008 (0.934–1.187)	1.012 (0.934–1.096)	1.029 (0.966–1.096)	1.039 (0.957–1.128)	0.973 (0.902–1.050)
High Servings	1.072 (0.986–1.166)	1.055 (0.964–1.154)	1.126 (1.051–1.206) **	1.088 (0.998–1.186)	0.996 (0.915–1.084)
*Model 4*					
None Servings	1	1	1	1	1
Medium Servings	1.039 (0.964–1.120)	0.992 (0.913–1.078)	1.028 (0.963–1.096)	0.994 (0.912–1.083)	0.926 (0.854–1.004)
High Servings	1.076 (0.989–1.170)	1.042 (0.948–1.144)	1.133 (1.054–1.217) **	1.025 (0.936–1.123)	0.927 (0.845–1.016)
**SSB Frequency/week**					
*Model 1*					
None Frequency	1	1	1	1	1
Medium Frequency	1.078 (1.008–1.153) *	1.062 (0.988–1.141)	1.036 (0.980–1.097)	0.983 (0.915–1.057)	1.004 (0.938–1.074)
High Frequency	1.067 (1.005–1.132) *	0.995 (0.933–1.061)	1.092 (1.039–1.147) ***	1.347 (1.268–1.431) *	0.946 (0.891–1.005)
*Model 2*					
None Frequency	1	1	1	1	1
Medium Frequency	1.056 (0.987–1.130)	1.035 (0.962–1.113)	1.039 (0.982–1.100)	0.972 (0.903–1.046)	0.997 (0.932–1.067)
High Frequency	1.042 (0.980–1.107)	1.019 (0.954–1.089)	1.102 (1.048–1.158) ***	1.034 (0.971–1.101)	0.966 (0.909–1.028)
*Model 3*					
None Frequency	1	1	1	1	1
Medium Frequency	1.029 (0.950–1.114)	1.023 (0.941–1.113)	1.014 (0.949–1.084)	0.991 (0.907–1.081)	0.991 (0.916–1.073)
High Frequency	1.055 (0.981–1.134)	1.015 (0.940–1.097)	1.092 (1.028–1.159) *	1.064 (0.986–1.149)	0.990 (0.920–1.065)
*Model 4*					
None Frequency	1	1	1	1	1
Medium Frequency	1.033 (0.952–1.120)	0.995 (0.913–1.084)	1.009 (0.943–1.079)	0.961 (0.879–1.051)	0.954 (0.880–1.035)
High Frequency	1.063 (0.985–1.148)	0.992 (0.915–1.076)	1.094 (1.027–1.165) *	1.049 (0.968–1.136)	0.963 (0.892–1.040)

SSB, sugar-sweetened beverage; * *p* < 0.05; ** *p* < 0.01; *** *p* < 0.001; Model 1: without adjustment; Model 2: adjusted for age, sex, and residence; Model 3: further adjusted for maternal education, paternal education, family income, screen time, and physical activity; Model 4: further adjusted for meat and fried food for overweight, obesity, and abdominal obesity; and meat, fried food, height, and BMI for blood pressure.

## References

[1] Shields M. (2006). Overweight and obesity among children and youth. Health Rep..

[2] Sun H.P., Ma Y.N., Han D., Pan C.W., Xu Y. (2014). Prevalence and trends in obesity among China’s children and adolescents, 1985–2010. PLoS ONE.

[3] Dong B., Wang Z., Song Y., Wang H.J., Ma J. (2015). Understanding trends in blood pressure and their associations with body mass index in Chinese children, from 1985 to 2010: A cross-sectional observational study. BMJ Open.

[4] Chen X., Wang Y. (2008). Tracking of blood pressure from childhood to adulthood: A systematic review and meta-regression analysis. Circulation.

[5] Singh A.S., Mulder C., Twisk J.W., van Mechelen W., Chinapaw M.J. (2008). Tracking of childhood overweight into adulthood: A systematic review of the literature. Obes. Rev..

[6] Kelly A.S., Barlow S.E., Rao G., Inge T.H., Hayman L.L., Steinberger J., Urbina E.M., Ewing L.J., Daniels S.R. (2013). Severe obesity in children and adolescents: Identification, associated health risks, and treatment approaches. Circulation.

[7] Daniels S.R., Pratt C.A., Hayman L.L. (2011). Reduction of risk for cardiovascular disease in children and adolescents. Circulation.

[8] Koplan J.P., Dietz W.H. (1999). Caloric imbalance and public health policy. JAMA.

[9] Richelsen B. (2013). Sugar-sweetened beverages and cardio-metabolic disease risks. Curr. Opin. Clin. Nutr. Metab. Care.

[10] Vos M.B., Kaar J.L., Welsh J.A., van Horn L.V., Feig D.I., Anderson C.A., Patel M.J., Cruz M.J., Krebs N.F., Xanthakos S.A. (2016). Added sugars and cardiovascular disease risk in children: A scientific statement from the American Heart Association. Circulation.

[11] Kit B.K., Fakhouri T.H., Park S., Nielsen S.J., Ogden C.L. (2013). Trends in sugar-sweetened beverage consumption among youth and adults in the United States: 1999–2010. Am. J. Clin. Nutr..

[12] Brand-Miller J.C., Barclay A.W. (2017). Declining consumption of added sugars and sugar-sweetened beverages in Australia: A challenge for obesity prevention. Am. J. Clin. Nutr..

[13] Sánchez-Pimienta T.G., Batis C., Lutter C.K., Rivera J.A. (2016). Sugar-sweetened beverages are the main sources of added sugar intake in the Mexican population. J. Nutr..

[14] Aburto T.C., Pedraza L.S., Sánchez-Pimienta T.G., Batis C., Rivera J.A. (2016). Discretionary foods have a high contribution and fruit, vegetables, and legumes have a low contribution to the total energy intake of the Mexican population. J. Nutr..

[15] World Health Organization (WHO) (2015). Guideline: Sugars Intake for Adults and Children.

[16] Shang X.W., Liu A.L., Zhang Q., Hu X.Q., Du S.M., Ma J., Xu G.F., Li Y., Guo H.W., Du L. (2012). Report on childhood obesity in China (9): Sugar-sweetened beverages consumption and obesity. Biomed. Environ. Sci..

[17] He B., Long W., Li X., Yang W., Chen Y., Zhu Y. (2017). Sugar-sweetened beverages consumption positively associated with the risks of obesity and hypertriglyceridemia among Children aged 7–18 years in South China. J. Atheroscler. Thromb..

[18] Bucher D.T.S., Keller A., Laure D.J., Kruseman M. (2016). Sugar-sweetened beverages and obesity risk in children and Adolescents: A systematic analysis on how methodological quality may influence conclusions. J. Acad. Nutr. Diet..

[19] Grimes C.A., Riddell L.J., Campbell K.J., Nowson C.A. (2013). Dietary salt intake, sugar-sweetened beverage consumption, and obesity risk. Pediatrics.

[20] Malik A.H., Akram Y., Shetty S., Malik S.S., Yanchou N.V. (2014). Impact of sugar-sweetened beverages on blood pressure. Am. J. Cardiol..

[21] Johnson R.J., Segal M.S., Sautin Y., Nakagawa T., Feig D.I., Kang D.H., Gersch M.S., Benner S., Sanchez-Lozada L.G. (2007). Potential role of sugar (fructose) in the epidemic of hypertension, obesity and the metabolic syndrome, diabetes, kidney disease, and cardiovascular disease. Am. J. Clin. Nutr..

[22] Ludwig D.S., Peterson K.E., Gortmaker S.L. (2001). Relation between consumption of sugar-sweetened drinks and childhood obesity: A prospective, observational analysis. Lancet.

[23] Cunningham S.A., Zavodny M. (2011). Does the sale of sweetened beverages at school affect children’s weight?. Soc. Sci. Med..

[24] Laska M.N., Murray D.M., Lytle L.A., Harnack L.J. (2012). Longitudinal associations between key dietary behaviors and weight gain over time: Transitions through the adolescent years. Obesity.

[25] Payab M., Kelishadi R., Qorbani M., Motlagh M.E., Ranjbar S.H., Ardalan G., Zahedi H., Chinian M., Asayesh H., Larijani B. (2015). Association of junk food consumption with high blood pressure and obesity in Iranian children and adolescents: The CASPIAN-IV Study. J. Pediatr..

[26] Vilela S., Oliveira A., Pinto E., Moreira P., Barros H., Lopes C. (2015). The influence of socioeconomic factors and family context on energy-dense food consumption among 2-year-old children. Eur. J. Clin. Nutr..

[27] Andersen L.B., Arnberg K., Trolle E., Michaelsen K.F., Bro R., Pipper C.B., Molgaard C. (2016). The effects of water and dairy drinks on dietary patterns in overweight adolescents. Int. J. Food Sci. Nutr..

[28] DeBoer M.D., Scharf R.J., Demmer R.T. (2013). Sugar-sweetened beverages and weight gain in 2- to 5-year-old children. Pediatrics.

[29] Chen Y., Ma L., Ma Y., Wang H., Luo J., Zhang X., Luo C., Wang H., Zhao H., Pan D. (2015). A national school-based health lifestyles interventions among Chinese children and adolescents against obesity: Rationale, design and methodology of a randomized controlled trial in China. BMC Public Health.

[30] American Academy of Pediatrics (2001). Committee on public education. American academy of pediatrics: Children, adolescents, and television. Pediatrics.

[31] Fan M., Lyu J., He P. (2014). Chinese guidelines for data processing and analysis concerning the International Physical Activity Questionnaire. Zhonghua Liu Xing Bing Xue Za Zhi.

[32] Group of China Obesity Task Force (2004). Body mass index reference norm for screening overweight and obesity in Chinese children and adolescents. Zhonghua Liu Xing Bing Xue Za Zhi.

[33] Ma G.S., Ji C.Y., Ma J., Mi J., Yt S.R., Xiong F., Yan W.L., Hu X.Q., Li Y.P., Du S.M. (2010). Waist circumference reference values for screening cardiovascular risk factors in Chinese children and adolescents. Biomed. Environ. Sci..

[34] Mi J., Wang T.Y., Meng L.H. (2010). Development of blood pressure reference standards for Chinese children and adolescents. Chin. J. Evid. Based Pediatr..

[35] Yu P., Chen Y., Zhao A., Bai Y., Zheng Y., Zhao W., Zhang Y. (2016). Consumption of sugar-sweetened beverages and its association with overweight among young children from China. Public Health Nutr..

[36] Wang Y.C., Bleich S.N., Gortmaker S.L. (2008). Increasing caloric contribution from sugar-sweetened beverages and 100% fruit juices among US children and adolescents, 1988–2004. Pediatrics.

[37] Fiorito L.M., Mitchell D.C., Smiciklas-Wright H., Birch L.L. (2006). Dairy and dairy-related nutrient intake during middle childhood. J. Am. Diet. Assoc..

[38] Garriguet D. (2008). Beverage consumption of children and teens. Health Rep..

[39] Mirmiran P., Yuzbashian E., Asghari G. (2015). Consumption of sugar sweetened beverage is associated with incidence of metabolic syndrome in Tehranian children and adolescents. Nutr. Metab..

[40] Cohen L., Curhan G., Forman J. (2012). Association of sweetened beverage intake with incident hypertension. J. Gen. Int. Med..

[41] Sayon-Orea C., Martinez-Gonzalez M.A., Gea A., Alonso A., Pimenta A.M., Bes-Rastrollo M. (2015). Baseline consumption and changes in sugar-sweetened beverage consumption and the incidence of hypertension: The SUN project. Clin. Nutr..

